# Participation Mediates the Relationship Between Family Climate and Mental Well-Being Amongst Children with and Without Disability in a Cross-Sectional Swedish Registry-Based Study

**DOI:** 10.3390/ijerph23050632

**Published:** 2026-05-11

**Authors:** Lina Homman, Lilly Augustine, Mats Granlund

**Affiliations:** 1 Disability Research Division, Institute of Behaviour and Learning, Linköping University, 581 83 Linköping, Sweden; 2 CHILD, School of Education and Communication, Jönköping University, 551 11 Jönköping, Sweden; lilly.augustine@ju.se; 3 CHILD, School of Health and Welfare, Jönköping University, 551 11 Jönköping, Sweden; mats.granlund@ju.se

**Keywords:** mental health, participation, family climate, children, SEM mediation model, disabilities

## Abstract

**Highlights:**

**Public health relevance—How does this work relate to a public health issue?**

- The work investigates possible interrelations between participation and family climate regarding mental health in children, while also taking into account that mental health consists of both mental health problems and mental well-being;
- Children with disabilities are in focus as they face more obstacles on all three constructs of interest and are at increased risk of poor mental health.

**Public health significance—Why is this work of significance to public health?**

- The work indicates that participation mediated the relationship between family climate and mental well-being;
- Gender differences among children with disabilities are stronger while age differences are less pronounced concerning mental health.

**Public health implications—What are the key implications or messages for practitioners, policy makers and/or researchers in public health?**

- Participation and family climate are important factors impacting children’s mental health and a possible avenue for interventions and preventions;
- Disabilities need to be accounted for when planning interventions and preventions, as these children are at increased risk of mental health problems and indicate differences in the development of mental health problems.

**Abstract:**

Poor mental health (problems and well-being) among children, particularly those with disabilities, represents a significant individual and public health burden, with multifactorial origins including family environment and participation limitations. Family climate also impacts participation, suggesting the three structures may be interlinked. Furthermore, children with disabilities face additional obstacles increasing their vulnerability to poor mental health, such as limitations to participation. Therefore, the present study aimed to (1) investigate whether participation mediated the relationship between family climate and mental health and (2) whether possible relationships differed by disabilities. The Swedish Statistics (SCB) register on the conditions of life for children (barnULF) was utilised. This interview-based registry study employs a repeated cross-sectional design, where children are recruited yearly and invited to participate in the study. The present study used data from 2013–2019, resulting in 3676 children aged 10–18, of whom 510 reported some type of disability. Structural Equation Models (SEMs) mediation analyses were conducted to assess the possible mediating role of participation between family climate and mental health (well-being and problems separately), including models with and without covariates (age and gender). Multigroup analyses were conducted to assess whether children with and without disability differed. Results showed that participation significantly mediated the relationship between family climate and mental well-being but not mental health problems, amongst all children. Models including covariates indicated mediating models for both well-being and problems, but only amongst children without disabilities. However, model fits were poor. Implications of the study direct preventative strategies to focus on family climate as a route to improve mental well-being and highlight the importance of earlier strategies amongst children with disabilities and in particular girls.

## 1. Introduction

Mental health has often been considered as mental health problems or illness, with its opposite being merely the absence of mental health problems. A growing body of literature is changing this view, including mental well-being, not just as an opposite to mental health problems, but as a separate but interlinked construct, with distinct and similar risk and protective factors [1,2]. In contrast to viewing mental health problems and well-being as two ends of one spectrum, Keyes’ model of dual continua [1] hypothesised that mental health problems and well-being each consists of their own continua, where levels of one exists independently of the other, suggesting, for example, that an individual may have both high levels of mental health problems and high levels of well-being. Therefore, it is of importance to treat mental health problems and well-being as separate entities as they exist independently of one another, though they are likely to be interlinked. In addition, an increasing focus in research and applied practises on well-being is not only of benefit in itself but may also work preventatively towards mental health problems [3]. Mental well-being is not a novel concept but was described as early as 1946 by the WHO as: “a state of complete physical, mental, and social well-being and not merely the absence of disease or infirmity” [4]. Mental well-being includes aspects such as good cognitive functioning, sense of purpose and meaning, happiness, and the ability to manage everyday life, while mental health problems are the inability to do so and involve aspects of difficulties involving behaviours, cognition, or emotion regulation [5,6]; for an overview of the concept see [6]. The definitions are similar in children, where mental well-being is commonly related to reaching psychological, emotional and social milestones, while mental health problems are associated with distress and difficulties with functioning in psychological, social and emotional aspects [7].

A multitude of factors impact mental health in children, ranging from genetic factors to environmental factors, with a complex interplay between the two. One factor of particular importance due to its impact on several areas throughout childhood is the child’s family. The climate in which a child grows up—family climate, defined as the communication, cohesion, conflicts, interaction and relationships within the family [8,9]—has a lasting impact on the child’s current and future well-being. A good family climate promotes mental well-being, while a poor one increases the risk of mental health problems [10]. Some of the family climate influential factors are stable, such as socioeconomic status and parents’ education, while other factors are more dynamic, such as peer relationships, family relationships, safety in the home environment, and parental monitoring [2,11].

Furthermore, family climate does not only directly impact mental health but may also do so indirectly through participation—a well-established promotor of mental well-being [12]. Family climate impacts activities promoting development, participation in social activities and mastery of skills [13,14,15]. In addition, aspects of family climate, such as parental time and support and family orientation [16], may promote or hinder participation, both through what may be available (financially, geographically, in the interests of caregivers), what may be encouraged/discouraged, and depending on the communication, support, and relationships between the child and caregiver. Family climate is therefore likely to impact participation. Participation is a particularly strong protective factor of well-being as it provides children with opportunities for social relationship building and can improve academic performance and develop a child’s identity [8]. Several studies [8,17] report that the relationship between well-being and participation, especially social participation and participation in physical activities, is relatively robust, while the relationship between mental health problems and participation is not as prominent [12,18].

However, participation is not easily available to everyone. One group of children where participation and mental well-being is consistently reported to be lower and where mental health problems are reported to be higher is children with disabilities [19]. Children with disabilities face more challenges and obstacles overall and have an increased risk of experiencing challenges to participation [20] and consequently attend fewer activities and are less involved when attending [12,20,21,22]. Thereby, these children cannot benefit to the same extent from the protective aspects of participation in relation to mental well-being. Importantly, having a disability does not necessarily directly reduce mental health; this happens in the interaction with other person factors and factors in the environment, in other words, relating to functioning. Disability can be measured as per the type of disability or diagnosis, and many studies do so, e.g., attention deficit hyperactivity disorder (ADHD) or autism spectrum disorder (ASD). However, many children with disabilities face the same challenges in interactions with the environment, independent of diagnosis [23]. Many children with a disability diagnosis related to problems with sensory, neuropsychological and mobility impairment have partly the same problems with everyday functioning [2,19] and thus may need similar or related interventions.

Although peer relations become more important with age for children [24], family climate factors remain very important throughout childhood. This is particularly true for children with disabilities [25], partly due to delayed development [24], but also due to the limitations a disability may pose in interactions with the surrounding environment outside of the family. Such limitations demand family-implemented environmental adaptations so as not to experience restrictions and limitations in participation in everyday activities [12,26,27]. Due to the increased load of adjustments and coordination of care, as well as an increase in mental load through, for example, worry for the child [28], the family climate is often impacted negatively as a consequence of the child’s disability. Parents of children with a disability often show an increased level of problems and decreased levels of physical and psychological well-being, resulting in parents having less time to promote a positive family climate [2], which might reduce parent–child communication [29] and negatively impact children’s participation and well-being. This opens up the possibility that the relationships between family climate and participation differ between children with and without disabilities.

As described, there are established relationships between family climate and mental health, as well as between participation and mental health, particularly mental well-being. The present study hypothesises that participation mediates some of the relationship between family climate and mental health, for example, through a good family climate’s promotion of healthy lifestyle choices; some of the promoting effect seen on well-being may be due to its promoting effects in other areas (such as participation). We further hypothesised that the relationships may differ depending on outcome: while a relationship is hypothesised for well-being, it is not necessarily so for problems as the link between participation and mental health problems is weak. The aim of the present study was therefore to investigate whether participation mediates the relationship between family climate and mental health and whether this differs between mental health problems and well-being. A second aim of the study was to understand whether the strength of these relationships differed between children with and without disabilities, as the latter group face more obstacles on all three constructs of interest and is at increased risk of poor mental health.

## 2. Materials and Methods

### 2.1. Study Design and Data Collection

The present study employs data from the Sweden Statistics (SCB) register on the *Living Conditions Survey of Children* (barnULF) [30]. Additional information on whether the children had a disability or not was collected through their caregivers (ULF/SILC) [31]. BarnULF is a sub-study of ULF/SILC, and children were recruited through their caregivers, who participated in ULF/SILC. ULF/SILC is a yearly repeated cross-sectional study conducted by SCB in Sweden, randomly recruiting participants aged 16 and over, stratified by age, gender and postcode. If participants in ULF/SILC were the caregivers of a child living in their household, they were asked whether their child could participate in barnULF. If consent was provided from caregiver and child, the child was included in barnULF. BarnULF has been performed yearly since 2001, but the present study utilised data from barnULF across the years of 2013–2019 as prior to 2013 no data on disability was collected. Children included in barnULF were 10–18 years of age in 2013 and 12–18 year of age from 2014 onwards. No other exclusion criteria were utilised.

Data collection was conducted through structured telephone interviews in Swedish for both children and caregivers. An employee of SCB performed the interview, trained in conducting the interview to standardise the procedure. Amongst children the interviews covered topics on school, family, participation, mental health, and relationships. The data is collected by SCB, a Swedish governmental body who manages population statistics and registry data on the Swedish population used by the government, researchers, media and the public. The data collected in barnULF is updated yearly to be of social relevance and is chosen by SCB based on current goals and interests. The questions included in the interviews have been pilot tested and assessed for quality control as well as missing data. Data was de-identified prior to being received by researchers. Data collection was in line with GDPR standards, and the present study has received ethical approval from the Swedish Ethical Board (Etikprövningsnämnden). The material has been published by the authors in previous articles using the same data but with a different study aim [31].

### 2.2. Participants

A total of 3682 children were included in the study. Disability information was not collected before 2013 or in 2017; these years were therefore excluded. Because mental health was the study’s outcome measure, missingness on mental health items was examined. The majority of the participants had provided data on all the mental health items (N = 3586 (97, 55%)), and only a minority had missing data on 1–5 items (1 item (N = 74, 2.01%), 2 items (N = 9, 0.24%) or 3–5 items (N = 7, 0.18%)). Five participants had not answered any mental health items, and one participant had answered only one item and was therefore excluded. This resulted in a total sample of 3676 participants, 1731 boys and 1946 girls, with a mean age of 14.41 (SD = 2.29). Disabilities, including hearing and visual impairment, mobility impairment, ADHD/ASD, Dyslexia/language impairment and other disability were reported by 510 children (269 boys and 241 girls) (see Table 1), and 82 of these children reported multiple conditions.

### 2.3. Material

In the barnULF, information that was collected conserved demographic information of school, family, friends, participation and mental health. Information on whether the child had a disability that impacted their daily life was collected in ULF/SILC by the caregiver. The scales used were based on the literature (e.g., [32]).

#### 2.3.1. Disability of the Child Impacting Their Daily Life

Disabilities included: dyslexia/dyscalculia/speech or language impairment, mobility impairment, ADHD/autism (Neuro Developmental Disorder, NDD), hearing impairment, visual impairment not possible to correct with aids, and other disability. The items were answered as yes or no. A binary disability variable was created to indicate whether the child had any disability.

#### 2.3.2. Mental Health

Based on the dual continua model [1], mental health was divided into (a) mental health problems (9 items) and (b) well-being (3 items). These items have been assessed by SCB to measure mental health problems and well-being and have been quality assessed by SCB. The items for mental health problems related to: poor sleep, headache, stomach-ache, tired in school, trouble falling asleep, stress, feeling down, difficulties concentrating, nervous and tense. Items were scored on a Likert scale ranging from 1 to 4 or 1 to 5, depending on the item, where 1 = absence of mental health problems/low level of problems and 4 or 5 = high prevalence of mental health problems/high level of problems. The items indicated an internal consistency alpha of 0.76. The indicators of well-being included 3 items: happy with oneself, good mood, and good general well-being. The items were scored on a 4- or 5-item Likert scale, where a score of 1 = absence of mental well-being/poor well-being, and a score of 4 or 5 = high prevalence of mental well-being/good well-being. The scale indicated an internal consistency alpha of 0.62. The mental health items were chosen based on thematic resemblance to well-established screening tools such as the Strengths and Difficulties Questionnaire (SDQ) [33] and Child Behavioural Checklist (CBCL) [34]. The items chosen for the present study have previously been used and shown to support a latent construct of mental health problems [35].

#### 2.3.3. Participation (Attendance)

The theoretical base for the selection of variables lies in the fact that social and physical activities are often shown to be the strongest participation predictor of our outcome measure—mental health—also were supported previously using the same data set [2]. Physical participation consisted of two items: (i) participation in organised physical activity and (ii) physical activity on one’s own accord; each answered as yes or no (where yes was coded as 1) and summed together (scale of 0–2). Social participation consisted of three items concerning the regularity of visiting friends, having friends at home, and meeting friends elsewhere; each item was measured on a 4-item Likert scale ranging from less often/never to every day. A variable for other activities led by an adult leader (such as music, scouts, etc.) was also included and scored as yes or no. The items indicated an internal consistency alpha of 0.47. Participation measures attendance and involvement across heterogeneous activity types and contexts [36,37]. Using a clinimetric approach, participation in one leisure activity might preclude participation in another, and activities may not generally correlate. Therefore, high internal consistency is not a reasonable expectation when the scale is intentionally designed to cover different activities [37,38].

#### 2.3.4. Family Climate

The selection of items relating to family climate included items related to areas indicated by the literature to be related to family climate: parental time, parental relationships, and parental conflict [9,16] as well as parental monitoring [39,40], and partaking in decisions [41]. The items included were: (i) whether the child experienced that their mother and father had time for them (scale from 1 to 5, where 1 = never and 5 = always), (ii) whether they got on/agreed with their mother and father (scale from 1 to 5, where 1 = never and 5 = always), (iii) whether they felt they were part of the decision-making process at home (scale of 1–4, where 1 = no never and 4 = yes always), (iv) whether their caregivers knew their whereabouts at most times (scale of 1–4, where 1 = never and 4 = always), and (v) whether they could confide in their caregivers when troubled or worried (yes or no item). The items had an internal consistency alpha of 0.64.

### 2.4. Statistical Analysis

The analysis was performed in three steps in line with common practise of SEM: (1) confirmatory factor analysis (CFA) of each latent construct in order to confirm that the hypothesised items for each construct are significant and thereby confirm construct stability, (2) SEM mediation models to assess the hypothesised mediation of participation between family climate and mental health, and (3) multigroup SEM mediation models to assess for possible differences between children with and without disabilities.

**Figure 1:**
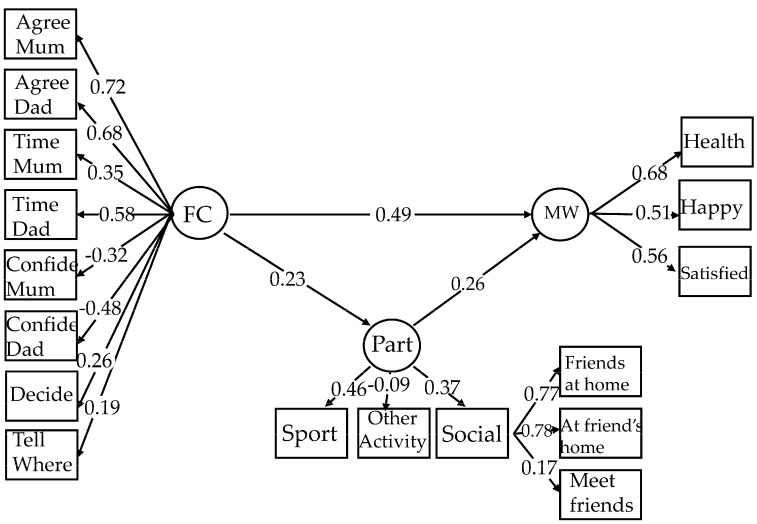
SEM Mediation model where family climate (FC) predicts mental well-being (MW), mediated by participation (Part). Only significant paths are shown. Standardised measures are shown.

In the initial step, four confirmatory factor analyses were conducted for each latent construct: family climate, participation, mental well-being, and mental health problems. For participation, 3 items were related to socialising with friends and were modelled as indicators onto a latent factor—social (see Figure 1).

In the second step, mediating SEMs were applied to assess our predictions of mediation; specifically, a mediation SEM was assessed where family climate was the independent variable, mental health the dependent, and participation the mediating variable. In models including covariates, age and gender were treated as covariates. Mediation models are of interest as they provide explanations of underlying functional mechanisms and how factors impact particular outcomes. SEMs are beneficial as they (i) assess the fit of data to a model, (ii) can estimate data as latent constructs, and iii) perform estimation of measurement error. SEMs measure structural relationships between variables and combine factor analyses, correlations and multiple regression analyses [42,43,44]. Parcelling was not necessary as the latent constructs were all defined by more than one indicator.

Two sets of SEM mediation analyses were performed to assess whether participation mediated the relationship between (1) family climate and well-being and (2) family climate and mental health problems. For family climate and well-being, an initial analysis assessed a mediation model with well-being as the outcome. For family climate and mental health problems, an initial analysis assessed a mediation model with mental health problems as the outcome. Covariates were then added to both mediation models, including age and gender, as mental health problems and well-being vary with age and by gender. The models were optimised in a stepwise approach by iteratively adding residual correlations between pairs of variables with the highest impact on model fit, repeated until there were no more such modifications which could make a meaningful improvement to the model fit.

To assess whether the mediation models differed between children with and without disabilities, multigroup SEM mediation models were performed by disability. Recent literature has found differences in measurement invariance between children with and without disabilities for mental health outcomes such as the SDQ and CBCL as well as the mental health outcomes used in barnULF [35]. Multigroup analysis is performed to assess whether the tested model remains invariant across respondent characteristics. Multigroup analysis involves comparing models with free and fixed parameters and comparing a more restrictive model with a less restrictive one to assess whether and which parameters differ across groups [45]. Models are run and compared in a predefined, stepwise order, consistent with how multigroup analysis is defined to be conducted [45]. The model’s run consists of: (M1) intercepts, factor loadings, and residual variance freed across groups and factor means fixed across groups (full measurement variance); (M2) intercepts and residual variance freed across groups while factor loadings and factor means are fixed across groups; (M3) residual variance freed while intercepts and factor loadings are fixed across groups while factor means are fixed at zero in the first group and freely estimated in the other; and (M4) factor means fixed at zero in the first group and freely estimated in the other groups, where intercepts, factor loadings and residual variance are fixed across groups (full measurement invariance). Models were compared using the DIFFTEST option in Mplus, presented as the difference in chi-square between models. Several fit indicators covering the most relevant aspects assessed whether the hypothesised models were a good fit of the data: RMSEA, CFI, TLI, and SRMR (see [46]). RMSEA assesses absolute fit indices, CFI assesses incremental fit index, TLI assesses elative reduction in misfit per degree of freedom, and SRMR assesses exact fit. RMSEA values below 0.06 indicate a good fit, SRMR values below 0.05, and TLI and CFI above 0.95. A satisfactory fit is indicated by RMSEA and SRMR below 0.08, TLI and CFI above 0.90 [47,48,49,50]. Statistical analysis was performed in STATA 14 and in Mplus 8.7 [51].

### 2.5. Missing Data

Only a small amount of missing data was observed on mental health (2.25%). Missing Completely At Random (MCAR) was excluded because the MCAR test was significant (*p* > 0.001). It is unlikely data is Missing Not At Random (MNAR), nor can we test for it. We therefore assumed the data was Missing At Random (MAR). Based on this assumption, multiple imputations (five) by chained equations were conducted using MICE.

## 3. Results

### 3.1. Fit of Latent Structures (CFA)

CFA of all constructs by the full sample and as multigroup analysis by disability is reported in Table 2. Relatively stable models were indicated across all analyses. All models fulfil the criteria for a good or acceptable fit, except participation, indicating a lower TLI, especially in the full sample. The models for children with disabilities indicated a somewhat better fit, especially for family climate and mental health problems and well-being. A very high fit concerning well-being in the disability group indicates that the latent structure is well adapted for this group. The consistently good fits of CFA supported that the constructs were suitable for SEM analysis.

### 3.2. SEM Mediation Analysis Model Fit

SEM mediation analysis fit indices for mental health problems and well-being by disability, with and without covariates, are presented in Table 3. Results indicated acceptable model fit for all models without covariates. When covariates were included, a relatively poor model fit was indicated. This was true for both the full sample and the disability sample, indicating that the covariates do not integrate well in the overall model.

### 3.3. Multigroup SEM Mediation Analysis

Two multigroup analyses of the mediation of mental health problems and well-being by disability are presented in Table 4. In each analysis, there are four models with increasing levels of restriction (ranging from M1 to M4), with the latter model compared with the previous one in order of numbers. Both multigroup analyses indicated significance between the least restrictive model and M2 and M3, but not M4, indicating that factor loadings, factor means, and intercepts (but not residual variance) significantly differed between children with and without disability in both mental health problems and well-being.

### 3.4. Overall Model Results

**Figure 2:**
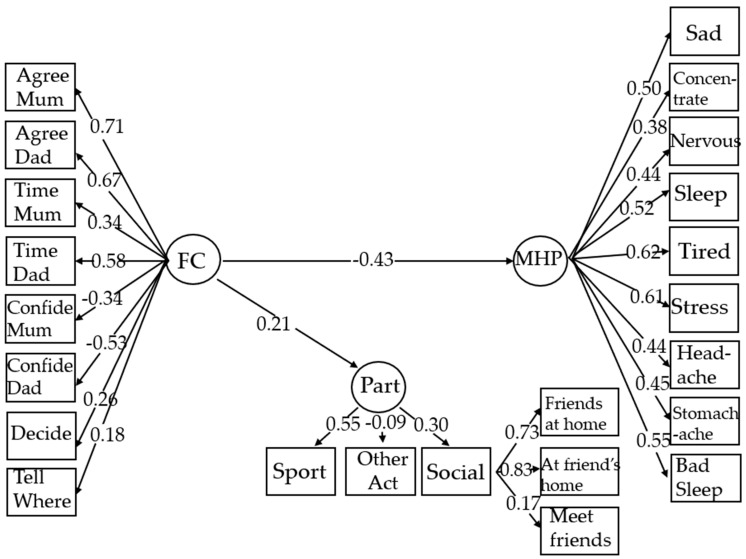
SEM Mediation model where family climate (FC) predicts mental health problems (MHPs) and is not mediated by participation (Part). Only significant paths are shown. Standardised measures are shown.

Path loadings of all models are presented in Table 5 and Table 6, and a mediation model with mental well-being as the outcome is depicted in Figure 1 (excluding covariates), and a mediation model with mental health problems as the outcome is depicted in Figure 2 (excluding covariates). Path loadings of well-being models, including a full model, with and without covariates, and models by disability, with and without covariates, are presented in Table 5. Path loadings of mental health problems models, including a full model with and without covariates, and models by disability, with and without covariates, are presented in Table 6.

In the SEM mediation model for well-being, the mediation models indicated significant factor loadings, except for other activities in the covariate model. Overall, most variables indicated stronger loadings amongst children with a disability but weaker path loadings. In well-being models including covariates, age significantly impacted well-being and participation, where an increase in age was associated with a decrease in well-being and participation. Gender significantly impacted well-being, where being female was associated with poorer well-being; however, there were no gender differences in participation. In all models, a significant mediation model was confirmed where (i) higher levels of family climate predicted higher levels of well-being and higher levels of participation, and (ii) higher participation predicted higher levels of well-being. However, this was not the case in models that included covariates and children with disabilities, in which no mediating relationship was observed.

In the SEM mediation model for mental health problems, most variables showed significant loadings, except for other activities. Path coefficients were weaker for children with disabilities. Family climate significantly predicted mental health problems and participation, where a better family climate predicted a decrease in mental health problems and an increase in participation. However, participation did not significantly predict mental health problems, suggesting that participation does not mediate the relationship between family climate and mental health problems.

Nevertheless, in mental health problems models including covariates, amongst children without a disability, a mediation model was supported where an increase in participation predicted a decrease in mental health problems. Age predicted mental health problems in children without a disability, but not in children with a disability, where an increase in age predicted an increase in mental health problems. Age also predicted participation, where an increase in age predicted a decrease in participation. Being female was significantly predictive of higher mental health problems. Gender and participation were only significantly associated in children without a disability, where being female was associated with lower levels of participation.

## 4. Discussion

The present study aimed to assess whether participation mediated the relationship between family climate and mental health (problems and well-being) in children with and without disabilities. We hypothesised that this would be the case, but more so for well-being than for mental health problems, given the stronger association between participation and well-being than between participation and mental health problems. The results did support our hypothesis—participation did mediate the relationship between family climate and well-being, but not between family climate and mental health problems. This was true among all children in models excluding covariates. The finding indicates that some of the relationship between family functioning and well-being is explained through participation; well-being may be increased by participation, but part of this is due to good family climate. However, in models including covariates, mediating relationships were found for both mental health problems and well-being, but only amongst children without a disability. However, the fits of these models were poor and should be interpreted with care.

SEM mediating models excluding covariates indicated that (a) better family climate predicted better mental well-being, lower mental health problems and increased levels of participation of the child, and (b) participation mediated the relationship between family climate and well-being (but not problems), where higher levels of participation improved well-being. This was true amongst both children with and without disabilities. The results are in line with previous literature on the positive relationship between participation and well-being in both children with and without disabilities [8,12] and add to the literature in explaining that part of this relationship is caused by family climate. Furthermore, the results strengthen the literature suggesting that while participation does improve well-being, it does not act as a buffer against mental health problems [52]. These findings support the growing body of literature arguing that mental health problems and well-being are separate constructs with separate risk and protective factors. The present study adds to previous findings by highlighting particularly important areas of children’s mental health and their interactions.

In models including covariates, a mediating relationship of participation was identified for both well-being and problems amongst children without a disability, but neither was identified amongst children with a disability. One possible explanation may lie in the fact that children with disabilities develop poor mental health earlier, despite following similar trajectories as children without a disability [12,53], a notion only visible when age was accounted for. This notion is also supported by the present findings in that while mental health problems increase with age amongst children without a disability, they did not significantly do so amongst children with a disability, arguably due to already being increased earlier. Previous literature also supports that girls with a disability develop problems earlier [54]. Gender differences observed are in line with the previous literature, supporting that girls report higher mental health problems and lower well-being as compared to boys [12,54]. The intersection between gender and disability is well documented (e.g., [12,54,55]), demonstrating that being female and having a disability may have cumulative adverse effects as both present obstacles.

As mentioned, we observed several differences between children with and without disabilities in the present study. Amongst children with a disability as compared to those without, stronger path loadings were observed for items on their latent constructs, but weaker path loadings between constructs were indicated. These results may indicate that disability effects are strong and that other intervention methods that directly involve the family may be necessary for this group; however, it is beyond the scope of the present study to draw any conclusions on this. Moreover, amongst children with a disability, the results suggest that age and gender are associated with participation and mental well-being to such a degree that the relationship is cancelled out in a mediation model. As mentioned earlier, poor mental health becomes observable at an earlier age amongst children with a disability, which may provide a partial explanation for the finding.

### 4.1. Limitations and Future Research

A limitation in the present study and an area for future research is the non-validated use of mental health items, particularly mental well-being, as the construct is measured with only three items. It should also be noted that these items relate only to emotional well-being, not to social or psychological well-being. The construct of participation could be improved as it only measures frequency of participation, not enjoyment or involvement in the activity. This is of importance as participation involves two dimensions, attendance in an activity and involvement in the activity if attending [36], where the former dimension is necessary for the latter. In addition, it is important to note that none of the items used were derived from standardised measures, and future studies should include variables such as family structure as variations may have been observed due to them. However, it is worth noting that the aim of the data is for Swedish registry purposes, not scientific or clinical, and neither is the aim to assess a clinical population. The results, therefore, need to be interpreted with this in mind.

Another factor worth considering is the difference between traits and states, where if traits are measured rather than states, a weaker relationship may be observed [19]. It is possible that the present study does measure traits concerning sociability more than states, and this fact should be considered when interpreting the results and taken into consideration in future studies. That this relationship was not present for children with a disability may be explained by the fact that children with a disability do participate to a lesser extent or under different circumstances and conditions, an area which would be interesting to explore in future research.

A further limitation is that the results need to be viewed in the light of COVID-19. The data in the present study were collected just prior to COVID-19. It is therefore not possible to generalise the results to the present day regarding the potential impact of COVID-19 on the areas highlighted in the present study: mental health, participation, and family climate. However, the study data are from Sweden, and COVID-19 did not impact the Swedish population to the same extent as in other parts of the world, as lockdowns were not utilised and schools were never closed. While the literature does indicate that self-harm in youth did increase during COVID-19 [56], there is not much else in the literature which indicates a larger impact on the current topics, nor is there evidence suggesting that things have not returned to baseline (prior to COVID-19). Future studies should address these shortcomings and assess whether the relationships found are consistent even today after COVID-19.

### 4.2. Implications

Two main implications from the present study are worth highlighting. First, for preventative and intervention purposes, the present study highlights the importance of improving mental well-being strategies focusing on helping families to promote participation. Secondly, children with disabilities, in particular girls, need early interventions to prevent poor mental health.

## 5. Conclusions

Conclusively, the present study highlights the importance of both family climate and participation for children’s mental well-being, and, in particular, their interlinkage. The results show that part of the promoting effects of participation on mental well-being are due to the effects of family climate on participation, indicating the importance of family climate in not only direct effects on the child, but also indirect. It further indicates that children with and without disabilities may need to be analysed separately in population studies since correlational patterns may be affected; e.g., age differences may be less pronounced and gender differences stronger among children with disabilities. Such differences may also be important to consider when planning interventions for increasing participation and well-being.

## Figures and Tables

**Table 1:** Age, gender and disability amongst children in barnULF 2013–2019, by year of data collection *.

	2013 (*n* = 724)	2014 (*n* = 677)	2015 (*n* = 553)	2016 (*n* = 547)	2018 (*n* = 607)	2019 (*n* = 569)	Total (*n* = 3676)
Age (Mean (SD))	13.75 (2.56)	13.81 (2.59)	14.87 (2.01)	14.92 (2.00)	14.64 (1.97)	14.88 (2.00)	14.41 (2.29)
Boys (% (N))	45.86 (332)	47.71 (323)	45.93 (254)	46.34 (253)	50.58 (307)	46.05 (262)	47.09 (17,316)
Disability (% (N))							
ADHD/ASD	3.18 (23)	2.81 (19)	3.25 (18)	3.66 (20)	5.77 (35)	4.22 (24)	3.78 (139)
Dyslexia etc	5.11 (37)	3.69 (25)	7.96 (44)	6.40 (35)	7.25 (44)	5.80 (33)	5.93 (218)
Visual impairment	1.80 (13)	2.22 (15)	0.54 (3)	0.37 (2)	0.82 (5)	1.58 (9)	1.28 (47)
Hearing impairment	1.25 (9)	0.89 (6)	1.81 (10)	0.73 (4)	0.99 (6)	1.76 (10)	1.22 (45)
Mobility impairment	0.97 (7)	0.74 (5)	2.53 (14)	1.46 (8)	0.66 (4)	0.88 (5)	1.17 (43)
Other	4.28 (31)	1.92 (13)	4.16 (23)	3.47 (19)	2.14 (13)	3.51 (20)	3.24 (119)
Total	16.57 (120)	12.26 (83)	20.25 (112)	16.09 (88)	17.63 (107)	17.75 (101)	13.87 (510)

* A very similar table including the same data has been published previously [4].

**Table 2:** Fit indices of CFA latent construct of family climate, participation and mental well-being and problems for full sample and multigroup CFA by disability.

		RMSEA	CFI	TLI	SRMR
Full sample	Family climate	0.057	0.960	0.925	0.035
	Participation	0.058	0.916	0.791	0.028
	Mental health problems	0.040	0.976	0.964	0.022
	Mental well-being	0.039	0.938	0.921	0.037
Disability	Family climate	0.046	0.968	0.958	0.039
	Participation	0.040	0.926	0.895	0.031
	Mental health problems	0.041	0.967	0.963	0.034
	Mental well-being	0.020	0.998	0.996	0.009

**Table 3:** SEM mediation analysis of mental health problems and mental well-being by disability, with and without covariates.

		RMSEA	CFI	TLI	SRMR
Mental health problems	Full model	0.035	0.919	0.905	0.036
	By disability	0.030	0.927	0.922	0.041
With covariates	Full model	0.045	0.859	0.836	0.137
	By disability	0.036	0.895	0.886	0.124
Mental well-being	Full model	0.039	0.938	0.921	0.037
	By disability	0.034	0.944	0.936	0.041
With covariates	Full model	0.052	0.880	0.847	0.154
	By disability	0.046	0.875	0.856	0.163

Covariates: gender and age. Good fit: RMSEA values of below 0.06, SRMR values below 0.05, TLI and CFI above 0.95. Acceptable fit: RMSEA and SRMR below 0.08, TLI and CFI above 0.90.

**Table 4:** Multigroup analysis of SEM mediation models by disability.

	χ^2^	Df	χ^2^ Difference	df	Sig
Mental health problems					
M1 (Full model variance)	1244.65	396	-	-	-
M2	1187.44	415	31.47	19	0.036
M3	1237.57	431	73.87	35	0.001
M4 (Full model invariance)	1240.41	439	15.19	8	0.056
Mental well-being					
M1 (Full model variance)	768.57	190	-	-	-
M2	752.94	203	23.74	13	0.034
M3	785.96	213	32.26	10	0.001
M4 (Full model invariance)	791.37	219	9.78	6	0.134

M1: full measurement variance, including intercepts, factor loadings, and residual variance freed across groups and factor means fixed across groups; M2: intercepts and residual variance freed across groups while factor loadings and factor mean are fixed across groups; M3: residual variance freed while intercepts and factor loadings are fixed across groups while factor means are fixed at zero in the first group and freely estimated in the other and; M4: full measurement invariance.

**Table 5:** Paths (path coefficients, standard errors, presented as standardized) in SEM mediation models (base model, for gender and for disability). Outcome: **well-being**.

	No Covariates			Covariates		
	Base Model	No Disability	Disability	Base Model	No Disability	Disability
Factor loadings on family climate						
Agree with mum	0.72 (0.01)	0.72 (0.01)	0.71 (0.03)	0.69 (0.01)	0.73 (0.01)	0.72 (0.03)
Agree with dad	0.68 (0.01)	0.68 (0.02)	0.66 (0.03)	0.71 (0.01)	0.67 (0.01)	0.65 (0.02)
Time mum	0.35 (0.01)	0.34 (0.01)	0.35 (0.02)	0.45 (0.02)	0.37 (0.01)	0.38 (0.02)
Time dad	0.58 (0.02)	0.57 (0.02)	0.58 (0.03)	0.60 (0.01)	0.59 (0.02)	0.61 (0.03)
Tell where	0.19 (0.02)	0.18 (0.02)	0.21 (0.02)	0.19 (0.02)	0.19 (0.02)	0.23 (0.02)
Confide mum	−0.32 (0.02)	−0.31 (0.02)	−0.38 (0.04)	−0.30 (0.02)	−0.31 (0.03)	−0.30 (0.05)
Confide dad	−0.48 (0.02)	−0.47 (0.03)	−0.49 (0.07)	−0.19 (0.03)	−0.47 (0.03)	−0.41 (0.06)
Decide	0.26 (0.02)	0.26 (0.02)	0.27 (0.02)	0.28 (0.02)	0.27 (0.02)	0.29 (0.02)
Factor loadings on participation						
Friend at home	0.78 (0.03)	0.76 (0.03)	0.83 (0.05)	0.83 (0.03)	0.82 (0.03)	0.89 (0.05)
At friend’s home	0.77 (0.03)	0.78 (0.03)	0.82 (0.04)	0.74 (0.03)	0.74 (0.03)	0.77 (0.04)
Friends other	0.17 (0.02)	0.17 (0.02)	0.18 (0.02)	0.19 (0.02)	0.18 (0.02)	0.19 (0.02)
Social	0.37 (0.04)	0.34 (0.04)	0.41 (0.06)	0.35 (0.03)	0.34 (0.04)	0.41 (0.06)
Sport	0.46 (0.05)	0.44 (0.05)	0.56 (0.08)	0.50 (0.04)	0.45 (0.04)	0.57 (0.07)
Other activity	−0.09 (0.04)	−0.09 (0.04)	n.s.	n.s.	n.s.	n.s.
Factor loadings on well-being						
Health	0.68 (0.02)	0.65 (0.02)	0.77 (0.03)	0.68 (0.02)	0.63 (0.02)	0.75 (0.03)
Happy	0.51 (0.02)	0.48 (0.02)	0.59 (0.03)	0.50 (0.02)	0.47 (0.02)	0.57 (0.03)
Satisfied	0.56 (0.02)	0.54 (0.02)	0.64 (0.03)	0.57 (0.02)	0.57 (0.02)	0.70 (0.03)
Family climate -> Well-being	0.49 (0.02)	0.51 (0.02)	0.40 (0.03)	0.47 (0.02)	0.48 (0.03)	0.38 (0.05)
Family climate -> Participation	0.23 (0.04)	0.21 (0.04)	0.17 (0.04)	0.20 (0.03)	0.23 (0.04)	n.s.
Participation -> Well-being	0.26 (0.04)	0.26 (0.04)	0.25 (0.05)	0.36 (0.05)	0.32 (0.05)	0.31 (0.09)
Age -> Well-being	n.a.	n.a.	n.a.	−0.08 (0.01)	−0.07 (0.01)	−0.06 (0.03)
Age -> Participation	n.a.	n.a.	n.a.	−0.12 (0.01)	−0.12 (0.01)	−0.14 (0.03)
Gender -> Well-being	n.a.	n.a.	n.a.	−0.30 (0.02)	−0.27 (0.02)	−0.36 (0.05)
Gender -> Participation	n.a.	n.a.	n.a.	n.s.	n.s.	n.s.

n.s. = non-significant; n.a. = not applicable (not applicable due to model non convergence resulting in model changes where social as a latent variable was removed); Gender (boy = 1, girl = 2).

**Table 6:** Paths (path coefficients, standard errors, presented as standardised) in SEM mediation models (base model, for gender and for disability). Outcome: **mental health problems**.

	No Covariates			Covariate		
	Base Model	No Disability	Disability	Base Model	No Disability	Disability
Factor loadings on family functioning						
Agree with mum	0.71 (0.01)	0.72 (0.01)	0.69 (0.03)	0.64 (0.02)	0.74 (0.02)	0.72 (0.03)
Agree with dad	0.67 (0.01)	0.68 (0.01)	0.63 (0.03)	0.60 (0.02)	0.65 (0.01)	0.63 (0.03)
Time mum	0.34 (0.02)	0.34 (0.02)	0.33 (0.02)	0.41 (0.02)	0.44 (0.03)	0.44 (0.03)
Time dad/	0.58 (0.02)	0.57 (0.02)	0.57 (0.03)	0.62 (0.02)	0.61 (0.02)	0.62 (0.03)
Tell where	0.18 (0.02)	0.18 (0.02)	0.20 (0.02)	0.19 (0.02)	0.19 (0.02)	0.21 (0.02)
Confide mum	−0.34 (0.03)	−0.34 (0.03)	−0.40 (0.04)	−0.32 (0.03)	−0.20 (0.03)	−0.19 (0.04)
Confide dad	−0.53 (0.03)	−0.52 (0.03)	−0.60 (0.07)	−0.47 (0.03)	−0.39 (0.04)	−0.40 (0.07)
Decide	0.26 (0.02)	0.26 (0.02)	0.26 (0.02)	0.21 (0.03)	0.34 (0.02)	0.35 (0.03)
Factor loadings on participation						
Friend at home	0.83 (0.04)	0.71 (0.03)	0.79 (0.05)	0.84 (0.03)	0.85 (0.04)	0.84 (0.07)
At friend’s home	0.73 (0.03)	0.83 (0.04)	0.88 (0.05)	0.75 (0.03)	0.73 (0.03)	0.69 (0.06)
Friends other	0.17 (0.02)	0.16 (0.02)	0.17 (0.02)	0.18 (0.02)	0.17 (0.02)	0.18 (0.02)
Social	0.30 (0.06)	0.28 (0.06)	0.33 (0.08)	0.40 (0.04)	0.19 (0.03)	0.13 (0.03)
Sport	0.55 (0.10)	0.52 (0.11)	0.64 (0.15)	0.43 (0.04)	0.28 (0.03)	0.20 (0.04)
Other activity	−0.09 (0.04)	−0.09 (0.04)	n.s.	n.s.	0.17 (0.04)	0.22 (0.10)
Factor loadings on mental health problems						
Sad	0.50 (0.01)	0.49 (0.02)	0.52 (0.03)	0.50 (0.02)	0.49 (0.02)	0.53 (0.03)
Concentrate	0.38 (0.02)	0.37 (0.02)	0.39 (0.03)	0.39 (0.02)	0.38 (0.02)	0.40 (0.03)
Nervous	0.44 (0.02)	0.43 (0.02)	0.44 (0.03)	0.45 (0.02)	0.44 (0.02)	0.46 (0.03)
Sleep	0.52 (0.02)	0.50 (0.02)	0.54 (0.04)	0.51 (0.02)	0.49 (0.02)	0.53 (0.04)
Tired	0.62 (0.02)	0.60 (0.02)	0.70 (0.04)	0.63 (0.02)	0.61 (0.02)	0.70 (0.04)
Stress	0.61 (0.02)	0.60 (0.02)	0.66 (0.04)	0.63 (0.02)	0.62 (0.02)	0.70 (0.03)
Headache	0.44 (0.02)	0.44 (0.02)	0.47 (0.03)	0.44 (0.02)	0.44 (0.02)	0.47 (0.03)
Stomach-ache	0.45 (0.02)	0.44 (0.02)	0.47 (0.03)	0.45 (0.02)	0.44 (0.02)	0.47 (0.03)
Poor sleep	0.55 (0.02)	0.53 (0.02)	0.54 (0.04)	0.54 (0.02)	0.53 (0.02)	0.55 (0.03)
Family climate -> Mental health problems	−0.43 (0.02)	−0.42 (0.02)	−0.39 (0.03)	−0.45 (0.02)	−0.48 (0.03)	−0.25 (0.08)
Family climate -> Participation	−0.21 (0.04)	−0.19 (0.04)	−0.16 (0.04)	−0.23 (0.04)	−0.33 (0.06)	n.s.
Participation -> Mental health	n.s.	n.s.	n.s.	−0.10 (0.04)	−0.26 (0.06)	n.s.
Age -> Mental health problems	n.a.	n.a.	n.a.	−0.30 (0.02)	−0.18 (0.02)	n.s.
Age -> Participation	n.a.	n.a.	n.a.	−0.28 (0.03)	−0.24 (0.01)	−0.32 (0.06)
Gender -> Mental health problems	n.a.	n.a.	n.a.	−0.27 (0.02)	−0.30 (0.02)	−0.30 (0.06)
Gender -> Participation	n.a.	n.a.	n.a.	n.s.	−0.10 (0.03)	n.s.

n.s. = non-significant; n.a. = not applicable (not applicable due to model non convergence resulting in model changes where social as a latent variable was removed), Gender (boy = 1, girl = 2).

## Data Availability

Data is unavailable due to privacy or ethical restrictions.

## Abbreviations

The following abbreviations are used in this manuscript:

SCB	Sweden Statistics
SEM	Structural Equation Modelling
WHO	World Health Organisation
ADHD	Attention Deficit Hyperactivity Disorder
ASD	Autism Spectrum Disorder
SDQ	Strength and Difficulty Questionnaire
CBCL	Child Behaviour Checklist
ULF/SILC	Living Conditions Survey
NDD	Neuro Developmental Disorder
